# Beyond the resistive index: value of color-coded duplex sonography parameters in kidney transplantation

**DOI:** 10.3389/fneph.2026.1718842

**Published:** 2026-02-04

**Authors:** Katharina Konzett, Sabrina Neururer, Martin Tiefenthaler

**Affiliations:** University Hospital Internal Medicine IV (Nephrology and Hypertensiology), Innsbruck Medical University, Innsbruck, Austria

**Keywords:** Banff classification, color-coded duplexsonography, kidney transplantation, non-invasive imaging biomarkers, renal allograft dysfunction, resistive index

## Abstract

Sonography is a key method in examining kidney transplants. Recent reports on the limited diagnostic value of an elevated resistive index (RI) in detecting rejection have cast doubt on the overall utility of color-coded duplex sonography (CCDS). This study evaluated additional CCDS parameters – percentage of vascularization (POV) and periphery vessel distance (PVD) – and investigated their association with histopathologic findings in allograft dysfunction. In a retrospective single-center study, 350 kidney transplant biopsies performed between 2013 and 2022 were analyzed. Standardized sonographic evaluation, including POV, PVD, and RI, was conducted at biopsy request. Histopathologic lesions were scored according to Banff criteria. Multivariable logistic regression identified independent predictors of abnormal CCDS parameters. Recipient age, severe tubular atrophy (ct), and arterial intimal fibrosis (cv) were independent negative predictors of POV > 50%. PVD ≥ 0.25 cm was associated with recipient age and moderate tubular atrophy (ct). RI showed no association with histopathologic lesions. These findings identify POV and PVD as non-invasive markers of chronic injury in kidney allografts, highlighting their potential adjunctive role in detecting parenchymal damage. As such, CCDS may support – but not replace – biopsy, which remains essential for establishing a definitive diagnosis in graft dysfunction.

## 1 Introduction

End-stage kidney disease (ESKD) represents a leading cause of global morbidity and mortality (1). Kidney transplantation is the preferred treatment for ESKD, offering substantial benefits in survival, quality of life, and cost-effectiveness compared to long-term dialysis (2–4). Since the pioneering success of human kidney transplantation by surgeon Joseph Murray in 1954 (5), there has been significant improvement in the short- and long-term survival of renal allografts from both living and deceased donors (6). However, in light of the rising prevalence of ESKD, driven by an ageing population and the increasing burden of diabetes and hypertension (7, 8), the persistent discrepancy between the number of transplantations performed and the number of patients on the waiting list underscores the urgent need to maximize long-term graft survival (9, 10).

In this context, it is of the highest interest to early diagnose renal allograft dysfunction (11). Histopathological examination via kidney biopsy is regarded as the gold standard for obtaining diagnostic information in cases of acute and chronic renal function decline in both native kidneys and transplants (12). Although renal biopsy is generally considered safe, it carries potential risks, including gross hematuria, bleeding requiring transfusion, and, in rare cases, graft loss or patient death (13). In anticoagulated patients, the increased bleeding risk often precludes timely biopsy, potentially delaying or compromising diagnostic accuracy (14). Furthermore, biopsy has inherent limitations such as sampling errors due to inadequate tissue, absence of cortex, advanced scarring, focal lesions, and diagnostic yield reductions from borderline changes or prior treatment (15).

The development of non-invasive diagnostic tools aims to improve the monitoring of kidney transplant recipients while reducing the risks associated with invasive procedures. Ultrasound, a widely available, cost-effective, and validated method, offers rapid assessment without radiation or nephrotoxic contrast agents (16). The Kidney Disease: Improving Global Outcomes (KDIGO) Clinical Practice Guideline for the care of kidney transplant recipients recommends sonography to evaluate allograft dysfunction (11), as it facilitates the detection of reversible causes including vascular complications, urinary obstruction, and perinephric fluid collections (17–20). Doppler sonography enables evaluation of vascular patency, flow direction, and complications such as renal artery stenosis, renal vein thrombosis, and arteriovenous fistulas. The American College of Radiology supports the use of Doppler ultrasound for both initial and follow-up assessment of transplant dysfunction. However, while Doppler-derived indices are useful for detecting large vessel complications, they are limited in assessing microvascular perfusion or structural parenchymal damage (21). Already in 1999, Venz et al. introduced color-coded duplex sonography (CCDS)-derived parameters for the assessment of perfusion. A reduced POV (≤ 55%) proved to be the best discriminator when chronic rejection was suspected (sensitivity 79%, specificity 87%). Tacrolimus nephrotoxicity showed not only a moderate elevation of the Doppler signal but also an increased PVD ≥ 3.9 mm and a normal POV. We applied these parameters in addition to the RI (22). As emphasized by current guidelines, biopsy remains the diagnostic gold standard, as imaging biomarkers, including emerging CCDS-derived parameters, have not yet been validated to reliably reflect underlying histopathology (21).

Against this background, the present study evaluated the relationship between the standardized CCDS parameters percentage of vascularization (POV), periphery vessel distance (PVD) and resistive index (RI), and histopathological findings, specifically Banff lesion scores, in renal allograft dysfunction.

## 2 Materials and methods

### 2.1 Study design and population

This single-center retrospective study was conducted at the Department of Internal Medicine IV, Nephrology and Hypertensiology, at the Medical University of Innsbruck. All adult kidney transplant recipients (≥ 18 years) who experienced allograft dysfunction and underwent diagnostic biopsy between January 1, 2013, and November 24, 2022, were included if the biopsy was performed at our department. Allograft dysfunction was defined as an unexplained acute or chronic increase in serum creatinine of ≥ 0.3 mg/dL, oliguria (urine output of < 500 mL/day or < 0.5 mL/kg/h), an increase in proteinuria (> 0.3 g/day), or the new onset of proteinuria. At our institution, no per-protocol or surveillance biopsies were performed during this period.

### 2.2 Biopsy procedure and histological assessment

Percutaneous ultrasound-guided core needle biopsy was performed using the tangential, extraperitoneal, retrorenal (TER) approach, a method developed at the Medical University of Innsbruck demonstrating excellent safety and efficacy (23). Histological assessment was conducted in-house until February 3, 2014, and subsequently at the Laboratory for Clinical Pathology in Hall or Innpath GmbH at the University Hospital Innsbruck. Specimens were evaluated according to the Banff classification of renal allograft pathology (24, 25), the current standard for transplant biopsy interpretation (26). The Banff system defines six diagnostic categories based on specific combinations of lesion scores, reflecting disease severity and chronicity (27). The scoring remained essentially unchanged, except that the latest version introduced C4d-negative humoral rejection. In our study population, no patients exhibited these lesions.

### 2.3 Ultrasound examination

**Figure 1:**
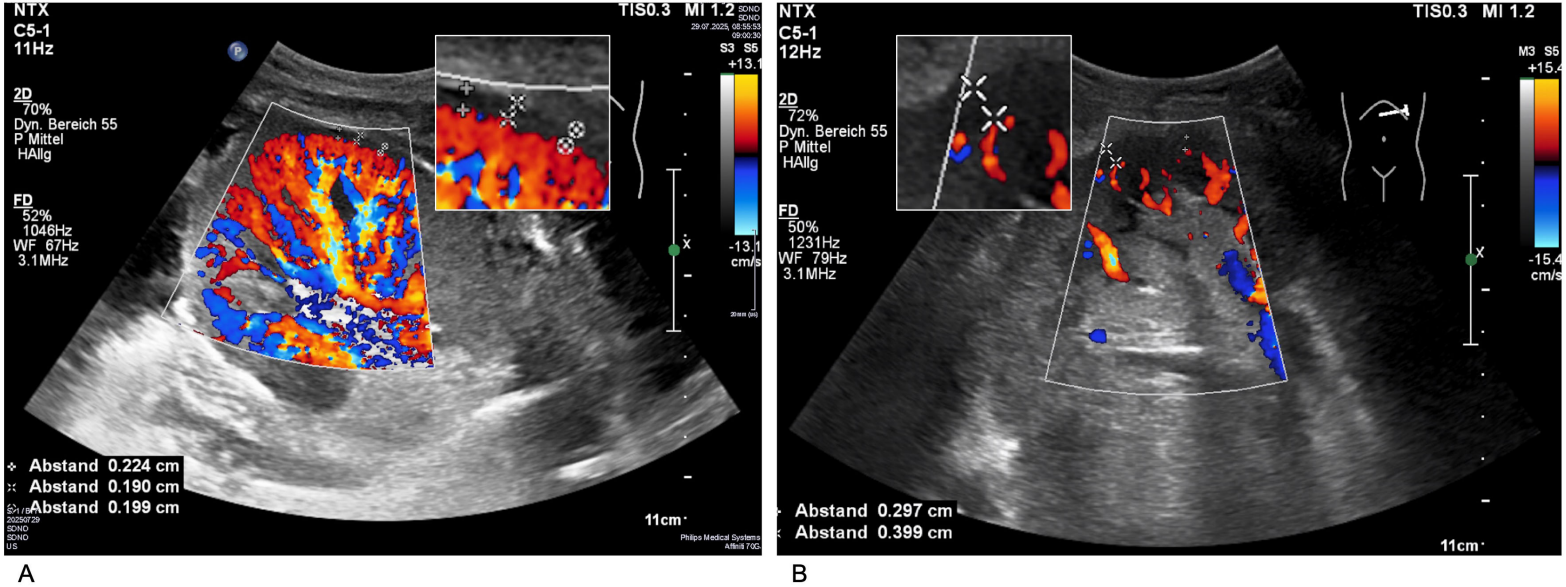
Ultrasound image of a well-perfused renal allograft with POV > 50% and PVD < 0.25 cm **(A)** versus image of a poorly perfused allograft with POV < 30% and PVD > 0.25 cm **(B)**. POV, percentage of vascularization; PVD, periphery vessel distance.

Unbiased sonography was conducted at the time of biopsy scheduling, preferably on the day prior to biopsy whenever feasible. Sonographic evaluation was performed using a standardized protocol with a convex transducer (1–6 MHz, Philips Affiniti 70). Grayscale images were obtained to assess transplant size, echotexture, hydronephrosis, and to measure renal parenchymal thickness. Perfusion analysis was conducted using CCDS at 3.1 MHz with a fixed color scale (–13.1 to +13.1 cm/s) and an average sensitivity of approximately 50%. POV, which describes the proportion of colored to non-colored renal parenchyma, was visually estimated from the most representative of several images and categorized as > 50% (physiological), 30–50% (indeterminate), or < 30% (reduced). The best frames from the recorded cine loops were selected for evaluation, and repeated measurements were performed. PVD was measured as the distance between the outermost visible vessel and the renal capsule, calculated as the mean of triplicate measurements, and classified as < 0.25 cm (normal) or ≥ 0.25 cm (pathological). Here as well, the mean of three measurements obtained from optimal cine loop frames was recorded. As a marker of vascular impedance, resistive index (RI) was determined by averaging Doppler spectra from three arcuate or interlobar arteries in the most ventrally positioned part of the kidney transplant, measured at < 60° insonation angle. Only spectra with PSV > 20 cm/s were included to avoid false measurements due to background noise. RI values were categorized as ≤ 0.75 (normal) or > 0.75 (pathological for patients < 65 years). Representative examples illustrating the different categories of POV and PVD are depicted in **Figure 1**.

### 2.4 Data collection

Relevant clinical, histological, and sonographic data were retrospectively extracted from the institutional electronic health records. General parameters included recipient age, sex, biopsy and ultrasound dates, and the number of biopsies performed per patient. Donor age was not included in analysis due to a previously reported lack of association with RI (27). Histological analysis included Banff lesion scores for the following parameters: glomerulitis (g), interstitial inflammation (i), total cortical inflammation (ti), tubulitis (t), intimal arteritis (v), arteriolar hyalinosis (ah), glomerular basement membrane double contours (cg), interstitial fibrosis (ci), tubular atrophy (ct), arterial intimal fibrosis (cv), mesangial matrix expansion (mm), peritubular capillaritis (ptc) and polyomavirus load level (pvl). In addition, main diagnoses (MD) were recorded. Sonographic parameters encompassed graft dimensions, parenchymal thickness, presence of hydronephrosis, and the CCDS metrics POV, PVD and RI.

### 2.5 Exclusion criteria

Patients were excluded if sonography was conducted outside standardized nephrology protocols. Biopsies with fewer than seven glomeruli or without at least one artery were excluded based on Banff criteria for diagnostic adequacy (24).

### 2.6 Statistical analysis

Statistical analysis was performed using IBM^®^ SPSS^®^ Statistics Version 29 (IBM Corp., Armonk, NY). Descriptive statistics and univariate analyses were initially conducted to explore the distribution of CCDS parameters and to identify potential associations with Banff lesion scores. Pearson’s chi-square test or Fisher’s exact test (for small sample sizes or expected frequencies < 5) was used for categorical comparisons. A two-sided p-value < 0.05 was considered statistically significant.

Variables with significant univariate associations were entered into multivariable regression models to identify independent predictors. To ensure parsimony and clinical interpretability, both full and reduced regression models were evaluated. For POV, the reduced ordinal logistic model demonstrated comparable explanatory power to the complete model (likelihood ratio chi-square (LR χ²) = 43.37 (reduced model) versus 49.60 (complete model); pseudo-R² = 0.063 versus 0.072), with improved model stability. For PVD, only the reduced binary logistic model reached statistical significance, while offering greater clarity and parsimony (p = 0.0071 (reduced model) versus 0.131 (full model); LR χ² = 15.92 versus 21.19; pseudo-R² = 0.033 versus 0.044). Reduced models were therefore used for final analysis. Results are reported as odds ratios (OR) or average marginal effects (AME), with 95% confidence intervals (CI) and p-values.

### 2.7 Statement of ethics

The study was conducted in accordance with the principles of the World Medical Association Declaration of Helsinki. The study protocol was reviewed and approved by the Ethics Committee of the Medical University of Innsbruck prior to initiation (approval number ECS 1258/2022). All patient data were pseudonymized, and data management complied with the European General Data Protection Regulation. Owing to the retrospective design, the ethics committee waived the requirement for written informed consent.

## 3 Results

### 3.1 Study population

**Figure 2:**
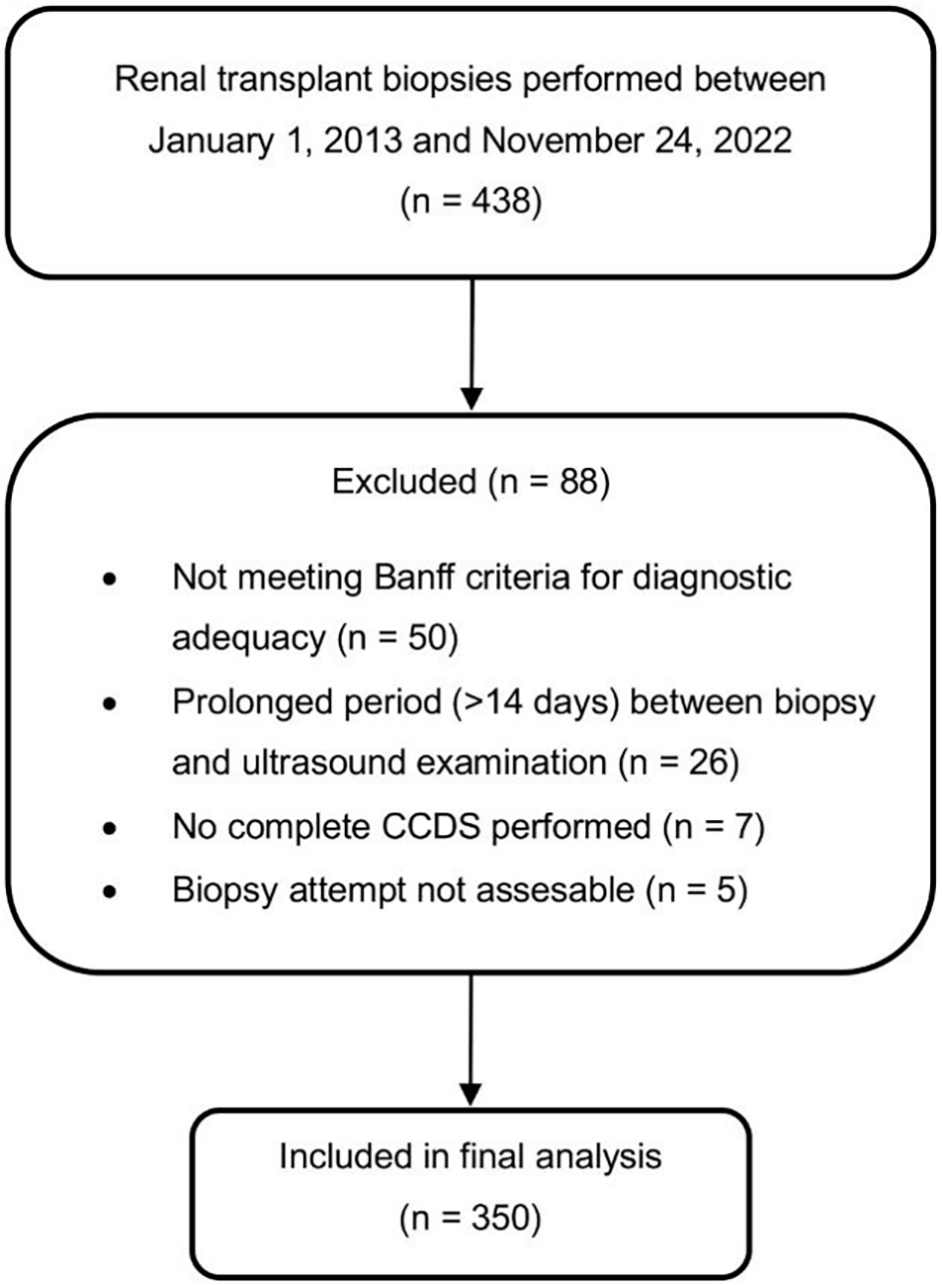
Study population. Flow chart of included and excluded kidney transplant biopsies. CCDS, color-coded duplex sonography.

Of the 438 renal allograft biopsies performed during the study period, 350 met the inclusion criteria (**Figure 2**). The most frequent reason for exclusion was failure to meet the Banff criteria for diagnostic adequacy (n = 50), followed by a biopsy-to-ultrasound interval exceeding 14 days (n = 26), incomplete CCDS examinations (n = 7), and unassessable samples due to necrosis (n = 5).

### 3.2 Descriptive results

Eligible biopsies were performed on 265 patients (66.6% male), with a median age of 52.7 years. The median interval between CCDS examination and biopsy was 1 day. Most patients (76.2%) underwent a single biopsy. POV > 50% was observed in 56.3% of cases, while 18.9% had a POV < 30%. PVD was ≥ 0.25 cm in 48.9%, and RI exceeded 0.75 in 48.3% of grafts. The most common Banff lesions were ci (85.4%) and ct (78.0%). Complete descriptive results are shown in **Supplementary Table S1**.

### 3.3 Univariate associations

Chi-square analyses revealed significant associations between POV and Banff lesion scores ah (p = 0.001), ci (p = 0.026), ct (p = 0.001), and cv (p < 0.001), along with the MD Chronic T cell-mediated rejection (TCMR) with intimal arteritis (p = 0.004) and the MD Active antibody-mediated rejection (ABMR) (p = 0.040). PVD was significantly associated with ct (p = 0.037) and the MD Active ABMR (p = 0.004) in univariate analysis. The RI showed no association with any histopathological lesion (**Table 1**).

**Table 1:** P-values from univariate analysis assessing the association between CCDS parameters and histopathological findings.

	CCDS parameter tested for association		
	POV	PVD	RI
Banff lesion score pvl (polyomavirus load level)	0.430	0.483	0.390
Banff lesion score g (glomerulitis)	0.068	0.119	0.801
Banff lesion score i (inflammation in non-scarred cortex)	0.390	0.372	0.418
Banff lesion score ti (total cortical inflammation)	0.586	0.442	0.288
Banff lesion score t (tubulitis in non-scarred cortex)	0.228	0.908	0.417
Banff lesion score v (endarteritis)	0.814	0.756	0.489
Banff lesion score ah (arteriolar hyalinosis)	0.001	0.259	0.308
Banff lesion score cg (chronic glomerulopathy)	0.088	0.055	0.499
Banff lesion score ci (interstitial fibrosis in cortex)	0.026	0.173	0.302
Banff lesion score ct (tubular atrophy in cortex)	0.001	0.037	0.544
Banff lesion score cv (arterial intimal fibrosis)	<0.001	0.485	0.364
Banff lesion score mm (mesangial matrix expansion)	0.094	0.075	0.070
Banff lesion score ptc (peritubular capillaritis)	0.655	0.055	0.097
MD Acute tubular injury	0.977	0.522	0.136
MD Polyomavirus allograft nephropathy	0.557	0.281	0.757
MD Acute TCMR without intimal arteritis	0.732	0.602	0.118
MD Chronic TCMR without intimal arteritis	0.312	0.947	0.203
MD Acute TCMR with intimal arteritis	0.703	0.285	0.409
MD Chronic TCMR with intimal arteritis	0.004	0.134	0.925
MD Borderline for acute TCMR	0.210	0.433	0.945
MD Active ABMR	0.040	0.004	0.554
MD Chronic ABMR	0.106	0.292	0.132

ABMR, antibody-mediated rejection; CCDS, color-coded duplex sonography; POV, percentage of vascularization; PVD, periphery vessel distance; RI, resistive index; TCMR, T cell-mediated rejection.

### 3.4 Multivariable analysis

Multivariable regression analysis identified recipient age, ct, and cv as independent negative predictors of a normal POV > 50% (**Table 2**). Each additional year of age was associated with a 0.68 percentage point decrease in the probability of a POV > 50% (AME = –0.0068, p < 0.001). Severe tubular atrophy (ct = 3) significantly reduced the likelihood of a normal POV by 37.6 percentage points (AME = –0.376, p = 0.001), whereas mild (ct = 1) and moderate (ct = 2) tubular atrophy had no statistically significant impact. In contrast, even mild arterial intimal fibrosis (cv = 1) was associated with a significantly lower probability of POV > 50% (AME = –0.166, p = 0.006), with a stepwise decline observed for moderate (cv = 2; AME = –0.153, p = 0.029) and severe arterial intimal fibrosis (cv = 3; AME = –0.217, p = 0.023).

**Table 2:** Effect estimates of clinical and histopathologic predictors on the probability of normal POV (> 50%): AME and p-values.

Predictor	Banff lesion score	AME	95% CI	P-value
recipient age	not applicable	–0.0068	[–0.0101, –0.0034]	<0.001
mild tubular atrophy	ct1	0.028	[–0.0998, 0.157]	0.664
moderate tubular atrophy	ct2	–0.079	[–0.232, 0.075]	0.315
severe tubular atrophy	ct3	–0.376	[–0.604, –0.148]	0.001
mild arterial intimal fibrosis	cv1	–0.166	[–0.285, –0.046]	0.006
moderate arterial intimal fibrosis	cv2	–0.153	[–0.289, –0.016]	0.029
severe arterial intimal fibrosis	cv3	–0.217	[–0.403, –0.030]	0.023

AME, average marginal effect; CI, confidence interval; POV, percentage of vascularization.

In the multivariate model for PVD, recipient age and ct emerged as independent predictors of an abnormal PVD ≥ 0.25 cm (**Table 3**). Older age was significantly associated with increased odds of PVD ≥ 0.25 cm (OR = 1.018, p = 0.032), corresponding to an average marginal increase of 0.42 percentage points per year of age (AME = +0.0042, p = 0.027). Moderate tubular atrophy (ct = 2) was associated with more than a twofold increase in the odds of pathological PVD compared to no atrophy (OR = 2.20, p = 0.018), and a 19 percentage point higher probability (AME = +0.19, p = 0.016). Severe tubular atrophy (ct = 3) showed a similarly elevated odds ratio (OR = 3.26) and a large marginal effect (AME = +0.28), but the association did not reach statistical significance (p = 0.098). A positive trend was also observed for TCMR with intimal arteritis (OR = 2.01; AME = +0.164), although this association did not achieve statistical significance (p = 0.095).

**Table 3:** Effect estimates of clinical and histopathologic predictors on the probability of high PVD (≥ 0.25 cm): OR, AME and p-values.

Predictor	Banff lesion score	OR	p-value (OR)	AME	95% CI	p-value (AME)
recipient age	not applicable	1.018	0.032	0.0042	[0.0005, 0.0080]	0.027
mild tubular atrophy	ct1	1.51	0.148	0.099	[–0.033, 0.231]	0.143
moderate tubular atrophy	ct2	2.20	0.018	0.190	[0.036, 0.345]	0.016
severe tubular atrophy	ct3	3.26	0.126	0.281	[–0.052, 0.613]	0.098
MD TCMR with intimal arteritis	not applicable	2.01	0.112	0.164	[–0.028, 0.356]	0.095

AME, average marginal effect; CI, confidence interval; MD, main diagnosis; OR, odds ratio; PVD, periphery vessel distance; TCMR, T cell-mediated rejection.

## 4 Discussion

This study demonstrates a significant association between the CCDS parameters POV and PVD with chronic histopathologic lesions in kidney allograft biopsies, supporting their potential utility as non-invasive markers of parenchymal graft injury. Among the investigated measures, POV emerged as the most sensitive parameter, showing a clear association with tubular atrophy (ct) and arterial intimal fibrosis (cv). Notably, even early stages of intimal fibrosis were associated with progressive reductions in POV, suggesting that POV may correlate both severity and extent of chronic microvascular injury. Consistent with these findings, PVD was also independently associated with tubular atrophy (ct), mirroring the parenchymal structural damage observed histologically. Recipient age was identified as a relevant predictor of abnormal POV and PVD, highlighting the importance of age-adjusted interpretation when assessing vascularization in kidney transplant recipients. Both CCDS parameters appear to reflect progressive microvascular remodeling and diminished allograft perfusion in older recipients.

A likely explanation for the observed associations between CCDS parameters and individual Banff lesion scores, rather than full diagnostic categories, lies in the composite nature of Banff diagnoses. Each diagnostic entity integrates multiple histologic components, which may occur in varying combinations and severities across biopsies. In contrast, CCDS parameters may primarily capture specific structural or hemodynamic alterations, such as vascular remodeling or parenchymal rarefaction, that are more directly linked to distinct histologic lesions than to broader diagnostic labels. This underscores the value of analyzing individual Banff lesion scores when evaluating imaging-histology correlations, as they provide a more nuanced reflection of the underlying pathophysiologic processes.

While univariate analyses had suggested associations between POV and additional histologic lesions, such as interstitial fibrosis, arteriolar hyalinosis, and both chronic and active forms of rejection, as well as between PVD and active ABMR, these did not persist in the multivariable models. This highlights the influence of confounding variables, particularly recipient age, and emphasizes the importance of adjusted analyses when interpreting these relationships.

The clinical utility of the RI in evaluating allograft dysfunction remains controversial. While early studies suggested that elevated RI values are linked to an increased risk of graft loss or recipient death (29) and may reflect tubulointerstitial or vascular injury (30), more recent evidence indicates that RI is influenced more by recipient-related factors – such as age and systemic hemodynamics – than by graft-specific pathology (28). In our study, RI showed no association with any histological lesion, supporting prior findings by Naesens et al. and Genkins et al., who reported poor correlations between RI and biopsy findings or transplant outcomes (28, 31). Although some reports linked elevated RI with adverse endpoints, delayed graft function or chronic allograft nephropathy (29, 32, 33), others failed to confirm these associations or emphasized intraindividual variation as a more meaningful metric (16). The American College of Radiology states that while RI values are often higher in dysfunctional allografts and may correlate with long-term graft survival or estimated glomerular filtration rate, they are neither sensitive nor specific for identifying the cause of dysfunction or for distinguishing between different histopathological processes (21). Our results add to the growing body of evidence suggesting that RI measurement plays a subordinate role in evaluating graft dysfunction. However, we acknowledge that intra-individual RI dynamics – despite also lacking specificity (28) – may still hold clinical relevance, as reported by Stigler et al. (16). Gilabert et al. described intra-individual RI changes following successful treatment of rejection, typically manifesting as a decrease in RI (34), although we have also observed paradoxical increases. It should be noted that the present analysis categorized RI as ≤ 0.75 or > 0.75 in accordance with local clinical practice. In contrast, several previous studies applied a cutoff of 0.80 (28, 29, 35), which may partially account for divergent findings.

To date, no other studies have systematically evaluated the association between the CCDS parameters POV and PVD and individual Banff lesion scores. Compared to RI, these parameters remain underutilized due to their absence from standardized protocols. However, several studies have demonstrated the diagnostic relevance of cortical perfusion markers. Venz et al. identified POV ≤ 55% as a sensitive and specific marker for chronic rejection, while elevated PVD was observed in cases of tacrolimus toxicity (22). Akl et al. and Trillaud et al. reported superior performance of power Doppler-based cortical perfusion grading over RI in predicting acute rejection and long-term graft function (36, 37). Syversveen et al. found significantly reduced perfusion intensity on color Doppler in grafts with moderate to severe fibrosis, correlating with estimated glomerular filtration rate (38). Similarly, Gao et al. showed that reduced interlobar PSV and EDV, but not RI, correlated with fibrotic histopathology (39). In contrast, Yoo et al. observed only weak or inconsistent associations between imaging biomarkers and Banff scores, emphasizing the complex pathophysiology of graft deterioration (40). A recent study comparing color Doppler and superb microvascular imaging found that the capsule-to-vessel distance correlated significantly with the chronic allograft index, a scoring system calculated using six histopathological lesions (interstitial inflammation, tubular atrophy, interstitial fibrosis, arterial fibrointimal thickening, glomerular mesangial matrix increase, and glomerular sclerosis), reinforcing the importance of perfusion-based imaging (41). Collectively, these findings highlight the promise of POV and PVD as adjunctive, non-invasive markers of parenchymal damage in renal allografts.

This study has several notable strengths, including a relatively large cohort, the use of a standardized sonographic protocol conducted by trained nephrologists, and strict adherence to Banff classification criteria. Although Banff criteria underwent revisions during the study period, these changes did not impact the present analysis, as they primarily concerned the definition of ABMR – most notably the allowance for C4d-negative cases – while the key histologic scoring parameters relevant to this study remained sufficiently consistent over time to allow for comparability (26). In addition, the short interval between imaging and biopsy (Median = 1 day) minimizes temporal bias and enhances the reliability of imaging-histology correlations. Furthermore, the study cohort is representative of the global risk population of kidney transplant recipients, with approximately two-thirds of biopsies performed in male patients. This sex distribution is consistent with prior reports, which attribute the male predominance in ESKD and renal transplantation to more rapid disease progression in men and sex-based disparities in access to advanced therapies, despite the higher overall prevalence of CKD in women (42–45).

However, several limitations of this study must be acknowledged. Its retrospective single-center design entails risks of selection bias, missing data, and residual confounding. Certain subgroup analyses, particularly those involving severe histologic lesions such as advanced tubular atrophy, may have been underpowered, as suggested by effect estimates that did not reach statistical significance despite clear trends. Similarly, while clinical experience and previous reports suggest a possible association between CCDS parameters and polyomavirus nephropathy, no significant association was observed in the present cohort. This may be due to the limited number of cases with biopsy-proven polyomavirus infection (n = 20). Additionally, current CCDS technology restricts measurement resolution: PVD values < 0.25 cm cannot be quantified, and POV > 50% cannot be further stratified. These constraints may mask subtle perfusion differences and limit the detection of more nuanced associations. Although polyomavirus nephropathy is often associated with preserved perfusion patterns, future technological advances and refined CCDS thresholds may improve diagnostic sensitivity. Moreover, Sonographic assessment, although performed by experienced examiners, remains operator-dependent and subject to intra-observer variability. Factors such as probe pressure, organ depth, gas interference, and the subjective estimation of POV may have contributed to measurement variability. Lastly, the absence of longitudinal follow-up precluded evaluation of the prognostic utility of CCDS parameters. Prospective multicenter studies are needed to validate POV and PVD as imaging biomarkers, define clinically meaningful thresholds, and explore their predictive value for graft outcomes over time.

In conclusion, this study identifies POV and PVD as valuable CCDS-derived markers for assessing kidney allograft changes, reflecting histologic features of chronic parenchymal injury, particularly tubular atrophy. The absence of association between RI and biopsy findings underscores the limited utility of RI as a standalone marker. While the integration of advanced sonographic parameters into post-transplant surveillance may offer a valuable, non-invasive window into allograft health and help guide individualized care, CCDS should be considered a complementary modality. Renal biopsy remains essential for establishing a definitive diagnosis in cases of graft dysfunction.

## Data Availability

The raw data supporting the conclusions of this article will be made available by the authors, without undue reservation.
