# Supplementary material for: Beyond the resistive index: value of color-coded duplex sonography parameters in kidney transplantation
Source: Front Nephrol. 2026 Feb 4;6:1718842. doi: 10.3389/fneph.2026.1718842 (PMC12913139; doi:10.3389/fneph.2026.1718842)
Supplement: Supplementary file 1 [file Table1.docx]

**Supplementary Table S1:** Descriptive Results

| Patients  male  female | 265  174 [65.7 %]  91 [34.3 %] |
| --- | --- |
| Number of performed biopsies  on male patients  on female patients | 350  233 [66.6 %]  117 [33.4 %] |
| Number of biopsies per patient  1  2  3  4  5 | 202 [76.2 %]  47 [17.7 %]  10 [3.8 %]  5 [1.9 %]  1 [0.4 %] |
| Age (years) | Median 52.7 [IQR 41.9–62.7] |
| Time between biopsy and ultrasound  examination (days) | Median 1 [IQR 1–3] |
| Length of the NTX (kidney transplant) (cm) | Median 11 [IQR 10–12] |
| Parenchymal thickness of the NTX (cm) | Median 1.5 [IQR 1.3–1.7] |
| Hydronephrosis  no hydronephrosis  existing hydronephrosis  Grade I  Grade II  Grade III  Grade IV | 328 [94 %]  21 [6 %]  13 [3.7 %]  7 [2 %]  1 [0.3 %]  0 [0 %] |
| POV  < 30 %  30-50 %  > 50 % | 66 [18.9 %]  87 [24.9 %]  197 [56.3 %] |
| PVD  < 0,25 cm  ≥ 0,25 cm | 178 [51.1 %]  170 [48.9 %] |
| RI  ≤ 0,75  > 0,75 | 172 [51.7 %]  161 [48.3 %] |
| Vmax (cm/s) | Median 32.1 [IQR 26.6–40] |
| Number of glomerula in the biopsy | Median 15 [IQR 10–21] |
| Number of arteries in the biopsy | Median 3 [IQR 2–4] |
| Banff lesion score pvl (polyomavirus load level)  0  1  2  3 | 330 [94.3 %]  9 [2.6 %]  3 [0.9 %]  8 [2.3 %] |
| Banff lesion score g (glomerulitis)  0  1  2  3 | 289 [82.8 %]  18 [5.2 %]  15 [4.3 %]  27 [7.7 %] |
| Banff lesion score i (inflammation non-scarred cortex)  0  1  2  3 | 190 [56.4 %]  98 [29.1 %]  44 [13.1 %]  5 [1.5 %] |
| Banff lesion score ti (total cortical inflammation)  0  1  2  3 | 172 [77.1 %]  32 [14.3 %]  16 [7.2 %]  3 [1.3 %] |
| Banff lesion score t (tubulitis in non-scarred cortex)  0  1  2  3 | 218 [62.5 %]  21 [6 %]  105 [30.1 %]  5 [1.4 %] |
| Banff lesion score v (endarteritis)  0  1  2  3 | 330 [94.3 %]  15 [4.3 %]  3 [0.9 %]  2 [0.6 %] |
| Banff lesion score ah (arteriolar hyalinosis)  0  1  2  3 | 212 [60.6 %]  75 [21.4 %]  37 [10.6 %]  26 [7.4 %] |
| Banff lesion score cg (chronic glomerulopathy)  0  1  2  3 | 274 [78.3 %]  28 [8 %]  18 [5.1 %]  30 [8.6 %] |
| Banff lesion score ci (interstitial fibrosis in cortex)  0  1  2  3 | 51 [14.6 %]  167 [47.7 %]  114 [32.6 %]  18 [5.1 %] |
| Banff lesion score ct (tubular atrophy in cortex)  0  1  2  3 | 77 [22 %]  182 [52 %]  82 [23.4 %]  9 [2.6 %] |
| Banff lesion score cv (arterial intimal fibrosis)  0  1  2  3 | 145 [41.8 %]  102 [29.4 %]  67 [19.3 %]  33 [9.5 %] |
| Banff lesion score mm (mesangial matrix expansion)  0  1  2  3 | 162 [46.3 %]  119 [34 %]  54 [15.4 %]  15 [4.3 %] |
| Banff lesion score ptc (peritubular capillaritis)  0  1  2  3 | 312 [89.1 %]  17 [4.9 %]  11 [3.1 %]  10 [2.9 %] |
| MD Acute tubular injury  yes  no | 83 [23.7 %]  267 [76.3 %] |
| MD Polyomavirus allograft nephropathy  yes  no | 20 [5.7 %]  330 [94.3 %] |
| MD Acute TCMR without intimal arteritis  yes  no | 29 [8.3 %]  321 [91.7 %] |
| MD Chronic TCMR without intimal arteritis  yes  no | 66 [18.9 %]  284 [81.1 %] |
| MD Acute TCMR with intimal arteritis  yes  no | 18 [5.1 %]  332 [94.9 %] |
| MD Chronic TCMR with intimal arteritis  yes  no | 8 [2.3 %]  342 [97.7 %] |
| MD Borderline (suspicious) for acute TCMR  yes  no | 21 [6 %]  329 [94 %] |
| MD Active ABMR  yes  no | 25 [7.1 %]  325 [92.9 %] |
| MD Chronic ABMR  yes  no | 34 [9.7 %]  316 [90.3 %] |

| *Data are presented as absolute and relative frequencies [valid percent] for nominal variables or median [IQR] for non-normally distributed metric variables (results rounded to one decimal place).* |
| --- |
